# Dual-task walking improvement with enhanced kinesthetic awareness in Parkinson’s disease with mild gait impairment: EEG connectivity and clinical implication

**DOI:** 10.3389/fnagi.2022.1041378

**Published:** 2022-11-30

**Authors:** Cheng-Ya Huang, Yu-An Chen, Ruey-Meei Wu, Ing-Shiou Hwang

**Affiliations:** ^1^School and Graduate Institute of Physical Therapy, College of Medicine, National Taiwan University, Taipei, Taiwan; ^2^Physical Therapy Center, National Taiwan University Hospital, Taipei, Taiwan; ^3^Division of Physical Therapy, Department of Rehabilitation, Shin Kong Wu Ho-Su Memorial Hospital, Taipei, Taiwan; ^4^Department of Neurology, National Taiwan University Hospital, College of Medicine, National Taiwan University, Taipei, Taiwan; ^5^Department of Physical Therapy, College of Medicine, National Cheng Kung University, Tainan, Taiwan; ^6^Institute of Allied Health Sciences, College of Medicine, National Cheng Kung University, Tainan, Taiwan

**Keywords:** Parkinson’s disease, gait disorder, dual task, attention, cortical connection, beta oscillation

## Abstract

Due to basal ganglia dysfunction, short step length is a common gait impairment in Parkinson’s disease (PD), especially in a dual-task walking. Here, we use electroencephalography (EEG) functional connectivity to investigate neural mechanisms of a stride awareness strategy that could improve dual-task walking in PD. Eighteen individuals with PD who had mild gait impairment walked at self-paced speed while keeping two interlocking rings from touching each other. During the dual-task walking trial, the participants received or did not receive awareness instruction to take big steps. Gait parameters, ring-touching time, and EEG connectivity in the alpha and beta bands were analyzed. With stride awareness, individuals with PD exhibited greater gait velocity and step length, along with a significantly lower mean EEG connectivity strength in the beta band. The awareness-related changes in the EEG connectivity strength of the beta band positively correlated with the awareness-related changes in gait velocity, cadence, and step length, but negatively correlated with the awareness-related change in step-length variability. The smaller reduction in beta connectivity strength was associated with greater improvement in locomotion control with stride awareness. This study is the first to reveal that a stride awareness strategy modulates the beta band oscillatory network and is related to walking efficacy in individuals with PD in a dual-task condition.

## Introduction

Parkinson’s disease (PD) is a neurodegenerative disorder that significantly affects motor function in these patients. The ability to walk is a primary concern in independent daily living for individuals with PD (Montero-Odasso et al., 2005). The situation of walking with a concurrent manual task (e.g., walking and carrying a plate filled with food) is common in daily life. For individuals with PD, especially those who can still walk without a walking aid, the ability to safely dual-task walk is essential for good quality of life. However, dual-task interference accentuates gait impairment (e.g., slower walking velocity, shortened stride length, and greater stride-to-stride variability), leading to a higher risk of falling (Kelly et al., 2012; van der Marck et al., 2014; Orcioli-Silva et al., 2020). In addition, the gait impairments in walking velocity, stride length, and stride-to-stride variability are worsened in the off-medication state (McNeely and Earhart, 2013; Orcioli-Silva et al., 2020; Stuart et al., 2020). For poor proprioceptive-motor integration, individuals with PD often have smaller stride lengths and swing amplitudes than they planned (Iansek et al., 2013), underlying a loss of neuronal response specificity in the cortico-basal ganglia-thalamo-cortical loop (Maschke et al., 2003; Vinding et al., 2019). The lack of kinesthetic awareness in PD can be alleviated by altering the cognitive state. For example, greater gait velocity and longer step length have been observed in individuals with PD when they use an awareness strategy (or attentional cuing) to focus on “taking big steps” (Canning, 2005; Baker et al., 2007; Lowry et al., 2010; Lohnes and Earhart, 2011). This awareness strategy has been shown to be effective for both single-task walking and dual-task walking (Canning, 2005; Baker et al., 2007; Lowry et al., 2010; Lohnes and Earhart, 2011).

Human walking is not a completely automatic process under subcortical control. Spatially distant cortical areas (e.g., frontal, parietal, temporal, and occipital areas) are activated and synchronized during human walking (Takakusaki, 2017; Wang and Choi, 2020). In heathy adults, cortical oscillatory activities in the alpha and beta bands are tuned to the attentional and sensorimotor processes (Engel and Fries, 2010; Başar, 2012), which are often modulated during single-task walking and various settings of dual-task walking (Beurskens et al., 2016; Protzak and Gramann, 2021). To compensate for basal ganglia dysfunction, individuals with PD often exhibit a broader range of activation and enhanced cortico-cortical connectivity during single-task walking and dual postural-motor tasks (Maidan et al., 2016a,b; Asher et al., 2021; Huang et al., 2022). The severity of gait impairment in individuals with PD is proportional to the synchronization strength in alpha and beta bands among frontal-motor-parietal areas (Asher et al., 2021). PD manifests with excessive phase synchronization in the alpha band during locomotion (Miron-Shahar et al., 2019). The use of an external cuing strategy for goal-directed control of PD gait results in a compensatory decrease in parieto-occipital alpha band activity, underlying the increased visual attention being paid to relevant information from the environment (Stuart et al., 2021; Tosserams et al., 2022). Compared to single-task walking, individuals with PD increased beta activity in response to dual-task walking that reflects the taxing of additional cognitive resources in more challenging tasks (Possti et al., 2021). There is also evidence of a strong frequency-correlation, showing a mechanistic linkage between abnormal beta oscillation and pathological motor processes in PD (Little and Brown, 2014). Individuals with PD who have bradykinesia, rigidity, or tremulous movement also demonstrate excessively high beta activity in the sensorimotor cortex and basal ganglia (Engel and Fries, 2010). The enhanced beta oscillations of regional and inter-regional activities are harmful to the coding of kinesthetic information prior to initiating a new movement, as greater beta oscillations in the motor loop favor persistence of the status quo (Engel and Fries, 2010).

Although the awareness strategy relies internally on cognitive mechanisms to improve gait control in PD (Iansek et al., 2013), the underlying neural mechanism of internal cueing, such as focusing on movement amplitude, remains unknown. However, it is expected to involve complex integrative functions with adaptive changes in large-scale cortical assemblages. Within the context of brain connectivity, this study aimed to investigate the effect of stride awareness in taking big steps in dual-task walking for individuals with PD. We focused on the awareness-related modulation of brain functional connectivity in the alpha and beta bands. In addition, this study aimed to assess the functional linkages between awareness-related changes in electroencephalography (EEG) functional connectivity and gait variables for dual-task walking. The study was designed to provide insight into the neurological mechanisms of stride awareness to improve dual-task walking in PD. Due to potential variations in sensorimotor processing, we hypothesized that individuals with PD would have less functional connectivity strength in alpha and beta subnetworks with better walking performance when they walked with an awareness strategy than when they walked without an awareness strategy. We also hypothesized that awareness-related changes in walking performance would be associated with awareness-related changes in functional connectivity, especially in the beta band.

## Materials and methods

### Participants

Eighteen individuals with PD (mean age: 63.1 ± 7.7 years) participated in this study. The inclusion criteria were: a diagnosis of idiopathic PD according to the United Kingdom PD Society Brain Bank clinical diagnostic criteria (Hughes et al., 1992), PD onset ≥40 years of age, and had a symptoms of mild gait impairment (no moderate or severe gait impairment). In the present study, mild gait impairment was defined as scores of 1 or 2 on item 2.12 (walking and balance) and item 3.10 (gait) of the MDS-sponsored Revision of the Unified Parkinson’s Disease Rating Scale (MDS-UPDRS). Item 2.12 and item 3.10 of the MDS-UPDRS are scored from 0 to 4 (Goetz et al., 2008). For item 2.12, the participants were asked about their walking and balance ability over the past week. A score of 1 on item 2.12 indicates the person had a symptom of slightly slow walking or leg drag, and never use a walking aid. A score of 2 on item 2.12 indicates the person occasionally used a walking aid, and did not need any help from other person. For item 3.10, the participants were asked to walk at least 10 meters, then turn around and return to the examiner. The measured items include stride amplitude, stride speed, height of foot lift, heel stride during walking, turning, and arm swing. A score of 1 or 2 on item 3.10 indicates the person can walk independently with minor or substantial gait impairment, and do not require an assistance device. Patients were excluded if they had a Mini-Mental State Examination (MMSE) score < 26, a history of brain surgery or other diseases and conditions that could influence balance ability, or a score > 2 on item 3.15 (postural tremor) and item 3.16 (kinetic tremor) of the MDS-UPDRS. A score > 2 on item 3.15 and item 3.16 indicates moderate or severe postural tremor or kinetic tremor in the hands, which may affect the accuracy of a manual task. Table 1 presents the demographic data and clinical characteristics of the participants. All procedures in this study were approved by the National Taiwan University Hospital Research Ethics Committee (Clinical Trial Registration No. NCT03298503), and all participants provided written informed consent.

**Table 1:** Patient demographics and characteristics.

	Mean/SD	Range
Age (in years)	63.1 ± 7.7	54.0 to 75.1
Sex (Male/Female)	9 M/9F	−
Disease duration (in years)	5.5 ± 1.8	3.5 to 8.0
Modified H&Y stage	2.3 ± 0.4	2 to 3
MMSE	28.3 ± 1.5	26 to 30
MDS-UPDRS motor scores	29.9 ± 5.0	21 to 38
item 2.12 (walking and balance)	1.16 ± 0.39	1 to 2
item 3.10 (gait)	1.32 ± 0.60	1 to 2

Values are presented as mean ± standard deviation unless otherwise indicated. Modified H&Y stage, Modified Hoehn and Yahr stage; MMSE, Mini-Mental Status Examination; MDS-UPDRS, Movement Disorder Society Unified Parkinson’s Disease Rating Scale.

The sample size was calculated based on data from a previous study (Lohnes and Earhart, 2011), which indicated that 17 participants would be sufficient to detect the effect of stride awareness on dual-task walking (Cohen’s d = 0.85, power = 0.9, α = 0.05).

### Experimental apparatus and data recording

**Figure 1:**
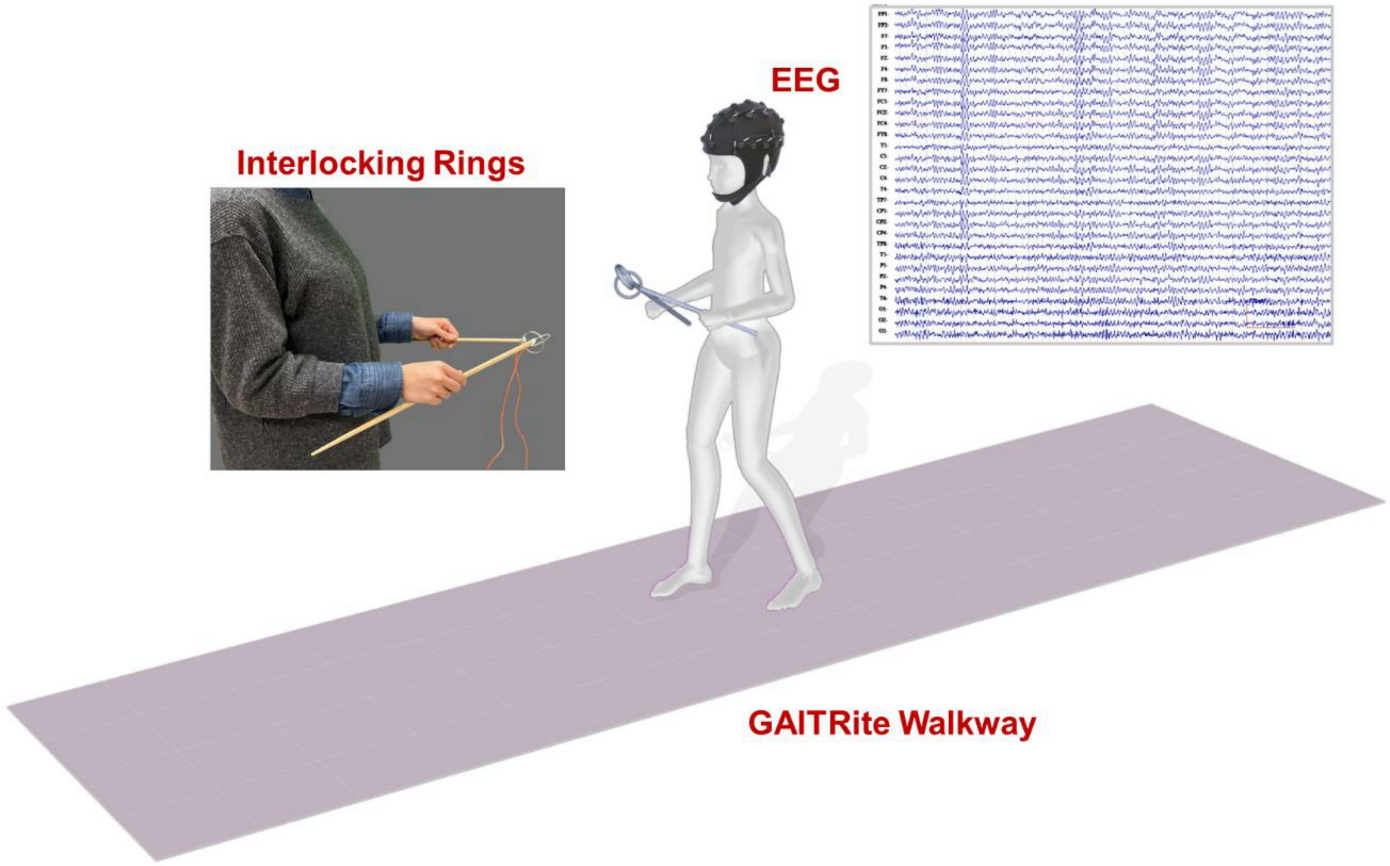
Diagram of the experimental setup.

The participants were instructed to walk on an electronic walkway (GAITRite, CIR Systems Inc., United States; sampling rate: 100 Hz) while controlling a pair of interlocking rings (Figure 1). The length of the GAITRite walkway was 5.20 m, with an active area of 4.27 m. To stabilize gait measures with the GAITRite walkway, the participants walked 2 m before and after the walkway for acceleration and deceleration. For the ring task, participants held two sticks and kept their elbows in 90° flexion. Attached to the end of each stick was a metal ring (diameter: 4 cm), and the two rings were interlocked (Beurskens et al., 2016; Huang et al., 2022). The participants were asked to prevent the two rings from touching each other. When the two rings touched, the computer recorded the event with an A/D card (USB-6221, National Instruments, United States; sampling rate: 1 kHz).

Cortical activities were recorded using a NuAmps 32-channel amplifier (NeuroScan Inc., United States). The EEG amplifier (height: 198 mm, width: 151 mm, depth: 40 mm, weight: 630 grams) was carried by participants in a backpack after proper fixation during locomotion like a mobile EEG system. The length of EEG wires outside the backpack was adjusted and fixed on participants’ upper back by several adhesive tapes to avoid movement artifacts caused by the movement of EEG wires. The cable connecting the EEG amplifier and recording computer was suspended to avoid interfering with walking. The similar method was used in a previous study for investigating gait initiation and stepping control (do Nascimento et al., 2005). The EEG electrodes (Fp1/2, Fz, F3/4, F7/8, FT7/8, FCz, FC3/4, Cz, C3/4, CPz, CP3/4, Pz, P3/4, T3/4, T5/6, TP7/8, Oz, and O1/2) were placed based on the 10–20 electrode system of the International Federation. The ground electrode was placed along the midline, ahead of Fz. To monitor vertical and horizontal eye movements and blinks, electrodes were placed above the left eyebrow, below the left eye, and horizontally on the outer canthi of both eyes. The impedances of all electrodes were ≤5 kΩ, and all electrodes were referenced to linked mastoids of both sides. EEG data were band-pass filtered at 0.1–100 Hz with a 1 kHz sampling rate. All behavioral data and EEG data were synchronized.

### Experimental conditions

All clinical assessments and dual-task walking examinations were performed in the morning on the same day, at least 12 h after the most recent administration of anti-parkinsonian medications (off-medication test) (Langston et al., 1992). There were two test conditions in this study: the non-awareness strategy (NAS) and the awareness strategy (AS). In the NAS condition, the participants were instructed to walk at their preferred speed and prevent the interlocking rings from touching. In the AS condition, the participants were instructed to pay attention to “taking big steps” while walking and prevent the interlocking rings from touching. The instruction was provided at the beginning of each test trial to emphasize the strategy application of non-awareness and awareness. The NAS condition was administered first to avoid carryover effects of walking awareness (Baker et al., 2007; Lowry et al., 2010). Two practice trials were conducted before eight testing trials in each experimental condition. Each test trial contained 7–9 walking steps (3–4 gait cycles per trial: 24–32 gait cycles per condition). Participants were asked to look straight ahead to avoid different postures or visual attention (e.g., looking at the rings or feet) during the test. The resting time was 30 s between two testing-trails and was 1 min between the NAS and AS conditions. Immediately following each test trial, participants were asked to rate the percentage of their attention that they felt had been directed towards walking in the NAS condition, or towards taking big steps in the AS condition, using an analogue scale (0–100%) to confirm the attentional allocation in each test trial (Canning, 2005).

### Data analysis

For the walking task, velocity, cadence, step length, and the coefficient of variation (CV) of step length were calculated. For the ring task, ring-touch time, which is the percentage of time in which the rings were touching in a walking trial, was calculated for each trial.

**Figure 2:**
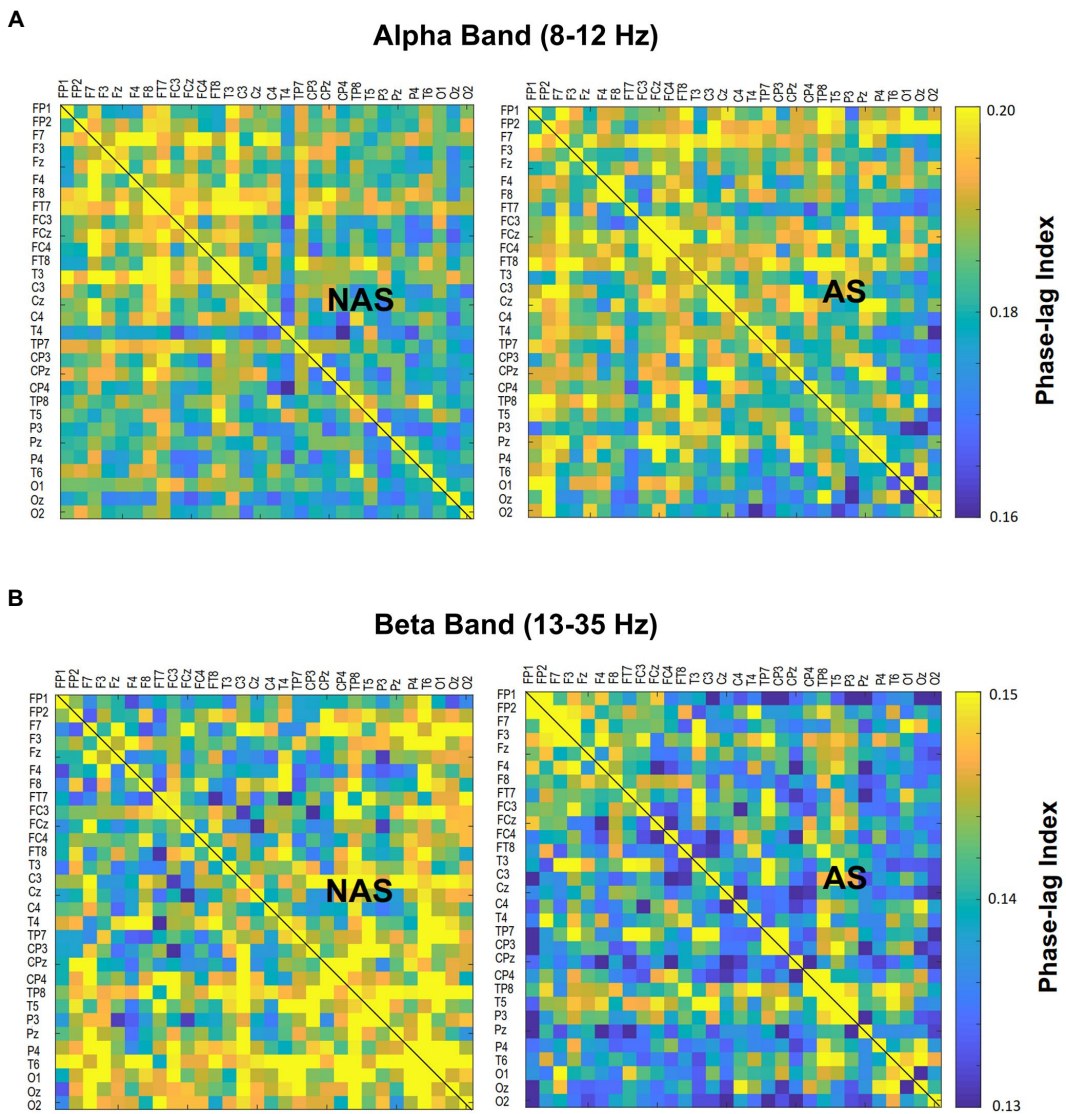
Contrast of population means in the PLI adjacent matrix in **(A)** alpha and **(B)** beta bands of all electrode pairs between the awareness strategy (AS) and non-awareness strategy (NAS) conditions. PLI, phase-lag index.

Using the NeuroScan software program (NeuroScan Inc., El Paso, TX, United States), the blinks and eye movements were corrected through the creation of bipolar vertical electrooculogram (EOG) channels by subtracting the activity in the infraorbitally placed electrode from the activity in the supraorbitally placed electrode, and the creation of bipolar horizontal EOG channels by subtracting the activity of the two electrodes placed on the outer canthi of both eyes. The conditioned EEG data were segmented with respect to gait cycles between two successive heel strikes by the same leg. The pre-processed EEG data were further conditioned with a phase-invariant impulse response (FIR) filter in the two frequency sub-bands: alpha (8–12 Hz) and beta (13–35 Hz). We did not analyze EEG data under 6 Hz (i.e., delta and theta bands) for potential contamination of movement artifacts during locomotion (Gwin et al., 2011). To characterize the strength of the inter-regional connectivity for the alpha and beta bands, a phase-lag index (PLI) was applied to EEG epochs of each gait cycle for all 30 electrode pairs in the alpha band and beta band, respectively (Figure 2). PLI was selected because this connectivity index is insensitive to common sources, such as volume conduction (Stam et al., 2007). Based on the Hilbert transformation (Stam et al., 2007), the PLI features the distribution asymmetry of phase differences in the instantaneous phases between a given pair of EEG epochs. If φ(t) is the phase difference, the PLI is defined as:$\;PLI=\left|E\left\{\mathrm{sgn}\left(\Delta \varphi \left(t\right)\right)\right\}\right|$, where *sgn* is a function that extracts the sign of a real number. The PLI functional connectivity was calculated using the HERMES function in Matlab (Niso et al., 2013). Calculation of PLI across all pairs of EEG channels resulted in a 30 × 30 PLI adjacent matrix. Within the 30 × 30 PLI adjacent matrix, a paired t-test with biased correction was used to correct non-normality and examine significant differences in the mean PLI of an electrode pair between the NAS and AS conditions. PLI matrices of all EEG electrode pairs were averaged to obtain the mean PLI (Alpha m-PLI and Beta m-PLI) in each condition. The 2-D matrix represented awareness-dependent differences in inter-regional connectivity, following the paired t-test to contrast PLIs for all electrode pairs between the NAS and AS conditions with biased correction. Both above-threshold (*p* < 0.05) and supra-threshold (*p* < 0.005) connections were highlighted to represent topological differences in connectivity strength between the NAS and AS conditions. From a statistical standpoint, the above-threshold edges denoted weaker AS-related differences in inter-regional connectivity. The supra-threshold edges denote relatively stronger AS-related differences in inter-regional connectivity. The EEG–EEG connectivity variables in each subject were estimated from EEG data of 24–32 artifact-free epochs (corresponding to gait cycles) by visual inspection for the NAS and AS conditions.

### Statistical analysis

On account of the non-normality of our data set, the Wilcoxon signed-rank test was used to examine the awareness effect on gait parameters, ring-touch time, and mean inter-regional EEG connectivity in the alpha (Alpha m-PLI) and beta (Beta m-PLI) bands. As variations in beta oscillation was a primary interest of this study, we further contrasted the mean PLIs of the NAS and AS conditions for above-threshold (Beta m-PLI_(*p* < 0.05)_) and supra-threshold (Beta m-PLI_(*p* < 0.005)_) connections in the subnetwork that were significantly increased (beta-connectivity enhancement) or decreased (beta-connectivity suppression) with the awareness strategy. In addition, Spearman correlation was used to examine the correlation of awareness-related changes (AS−NAS, △) between the m-PLI of the beta band and behavioral variables of the dual-task walking. The level of significance was set at *p* < 0.05. Signal processing and statistical analyses were completed using MATLAB v. R2018a (MathWorks, United States) and SPSS v. 21 (SPSS Inc., United States), respectively. All data are presented as the mean ± standard deviation.

## Results

All participants completed the experiment without adverse effect and falling. Based on an analogue scale, the participants reported that they allocated 40.2 ± 6.9% (31.1% − 52.9%) of total attention towards walking in the NAS condition and 73.9 ± 7.8% (62.0% − 87.5%) of total attention towards taking big steps in the AS condition. In the AS condition, the percentage of attention towards taking big steps in each test trial was above 60%, indicating the participants were actually attentive to take big steps during walking in the AS condition. Although we did not ask participants to rate the percentage of their attention that they felt had been directed towards interlocking rings in each trail, based on the task instructions of the NAS and AS conditions, we might assume that the attention was devoted to either walking or the ring task. Therefore, the participants about paid 59.8 ± 6.9% (47.1% − 68.9%) of total attention towards interlocking ring in the NAS condition and paid 26.1 ± 7.8% (12.5% − 38.0%) of total attention towards interlocking ring in the AS condition.

### Behavior performance

The Wilcoxon signed-rank test revealed that the AS condition had significantly greater gait velocity (*p* = 0.018) and step length (*p* = 0.002) than the NAS condition. We found no significant differences in cadence, step length CV, or ring-touch time (*p* > 0.05) between the NAS and AS conditions (Table 2).

**Table 2:** Gait velocity, cadence, step length, CV of step length, and ring touch-time in the NAS and AS conditions.

	NAS	AS	Statistics
Gait velocity (cm/s)	**85.33 ± 17.08**	**96.34 ± 19.01**	***z* = −2.373, *p* = 0.018**
Cadence (step/min)	89.66 ± 9.57	87.70 ± 9.03	*z* = −1.285, *p* = 0.199
Step length (cm)	**48.68 ± 6.57**	**55.32 ± 6.57**	***z* = −3.027, *p* = 0.002**
Step length CV (%)	5.60 ± 2.14	5.20 ± 1.53	*z* = −0.414, *p* = 0.679
Ring-touch time (%)	3.44 ± 3.23	5.19 ± 6.25	*z* = −1.372, *p* = 0.170

Values are presented as mean ± standard deviation. AS, awareness strategy; NAS, non-awareness strategy; CV, coefficient of variation. Bold values mean the significant difference between the NAS and AS conditions.

### EEG functional connectivity

**Figure 3:**
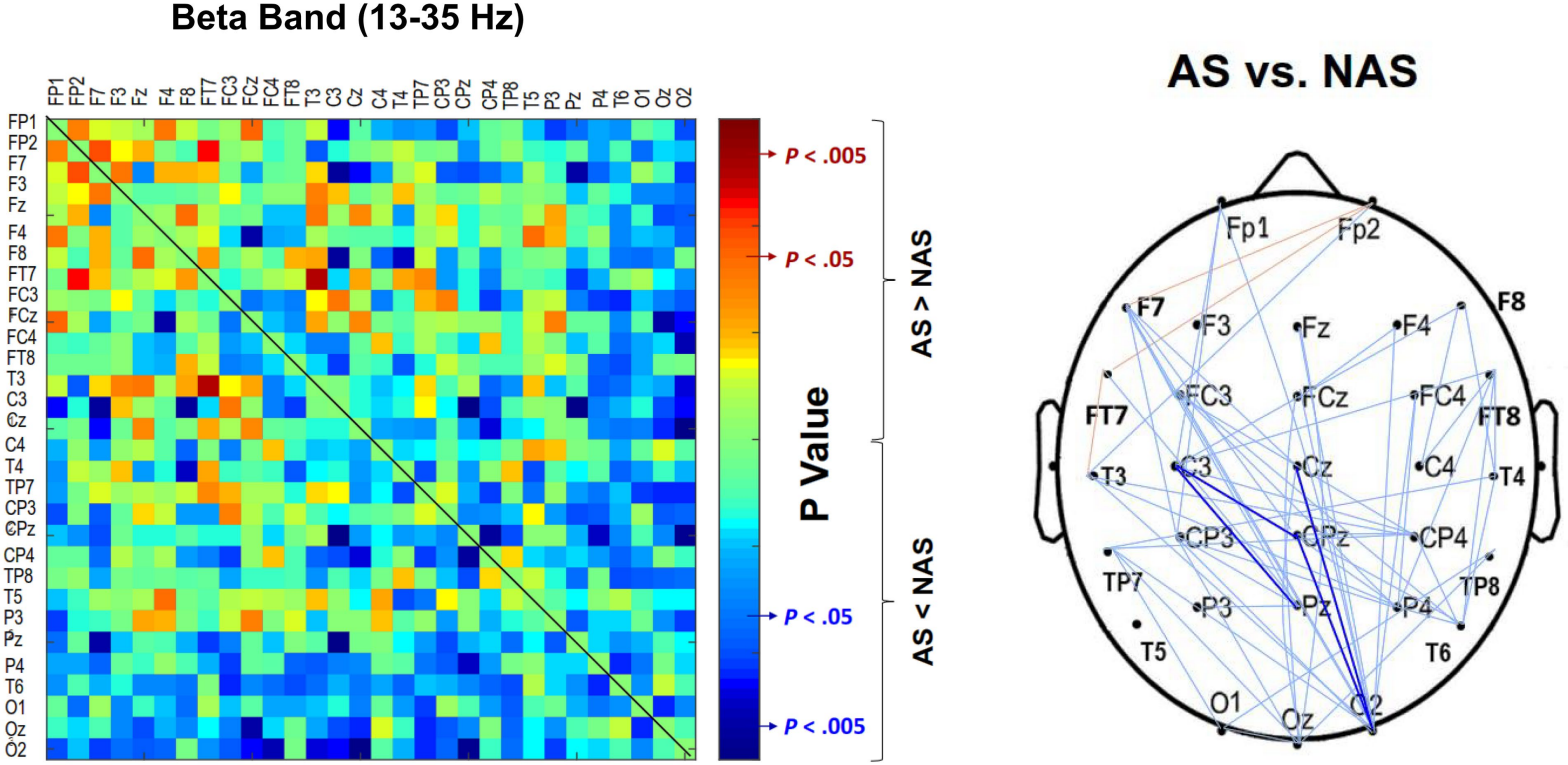
Adjacent matrices of *p*-values that contrast the PLI values of all electrode pairs between the AS and NAS conditions. A contrasting wiring diagram on the scalp shows the topological distributions of the above-threshold (*p* < 0.05) and supra-threshold connectivity (*p* < 0.005) in the beta band (13–35 Hz) tuned to stride awareness (NAS vs. AS). AS leads to widespread suppression of EEG beta connectivity in the fronto-centro-parietal-occipital areas. Dark blue line: AS < NAS, *p* < 0.005; light blue line: AS < NAS, *p* < 0.05; light red line: AS > NAS, *p* < 0.05. AS, awareness strategy; NAS, non-awareness strategy; PLI, phase-lag index.

The result of the Wilcoxon signed-rank test showed that the Alpha m-PLI was not subject to the awareness effect (*p* = 0.879), whereas Beta m-PLI was smaller in the AS condition than in the NAS condition (*p* = 0.016; Table 3A). Examined with a paired t-test with biased correction, Figure 3 shows the adjacent matrix of *p*-values that contrasts PLI values of electrode pairs in the beta band (the left plot) and topological differences in above-threshold (*p* < 0.05) and supra-threshold (*p* < 0.005) connections between the NAS and AS conditions (right plot). Relative to the NAS condition, most electrode pairs had less connectivity strength in the beta band in the AS condition across the fronto-centro-parietal-occipital areas. Only some frontal-area electrode pairs (e.g., Fp2-F7, Fp2-FT7, and FT7-T3) had enhanced above-threshold connectivity strength in the beta band in the AS condition. Regarding global suppression of connectivity strength in the beta band (electrode pairs represented by light and dark blue lines in Figure 3), the Beta m-PLI_(*p* < 0.05)_ and Beta m-PLI_(*p* < 0.005)_ were consistently smaller in the AS condition compared to the NAS condition (*p* < 0.001; Table 3B). In contrast, in the case of enhancement of above-threshold connectivity with stride awareness (electrode pairs represented by light red lines in Figure 3), the pooled m-PLI of the beta band (Beta m-PLI _(*p* < 0.05)_) for electrode pairs Fp2-F7, Fp2-F7, and FT7-T3 was not significantly different between the AS and NAS conditions (Table 3C).

**Table 3:** The m-PLI of the alpha and beta bands in the NAS and AS conditions for **(A)** all electrode pairs, **(B)** electrode pairs showing significantly suppressed connectivity with AS, and **(C)** electrode pairs showing significantly enhanced connectivity with AS.

	NAS	AS	Statistics
**(A) All connectivity pairs**			
Alpha m-PLI	0.188 ± 0.055	0.186 ± 0.048	*z* = −0.152, *p* = 0.879
Beta m-PLI	**0.144 ± 0.012**	**0.138 ± 0.012**	***z* = −2.417, *p* = 0.016**
**(B) Suppressed connectivity pairs**			
**Beta m-PLI**_**(*p* < 0.05)**_	**0.149 ± 0.010**	**0.134 ± 0.013**	***z* = −3.636, *p* < 0.001**
**Beta m-PLI**_**(*p* < 0.005)**_	**0.151 ± 0.020**	**0.132 ± 0.015**	***z* = −3.680, *p* < 0.001**
**(C) Enhanced connectivity pairs**			
**Beta m-PLI**_**(*p* < 0.05)**_	0.131 ± 0.024	0.138 ± 0.026	*z* = 0.675, *p* = 0.500

Values are presented as mean ± standard deviation. AS, awareness strategy; NAS, non-awareness strategy; m-PLI, mean phase-lag index; m-PLI_(*p* < 0.05)_, mean PLI from the electrode pairs of above-threshold (*p* < 0.05); m-PLI_(*p* < 0.005)_, mean PLI from the electrode pairs of supra-threshold (*p* < 0.005).

**Figure 4:**
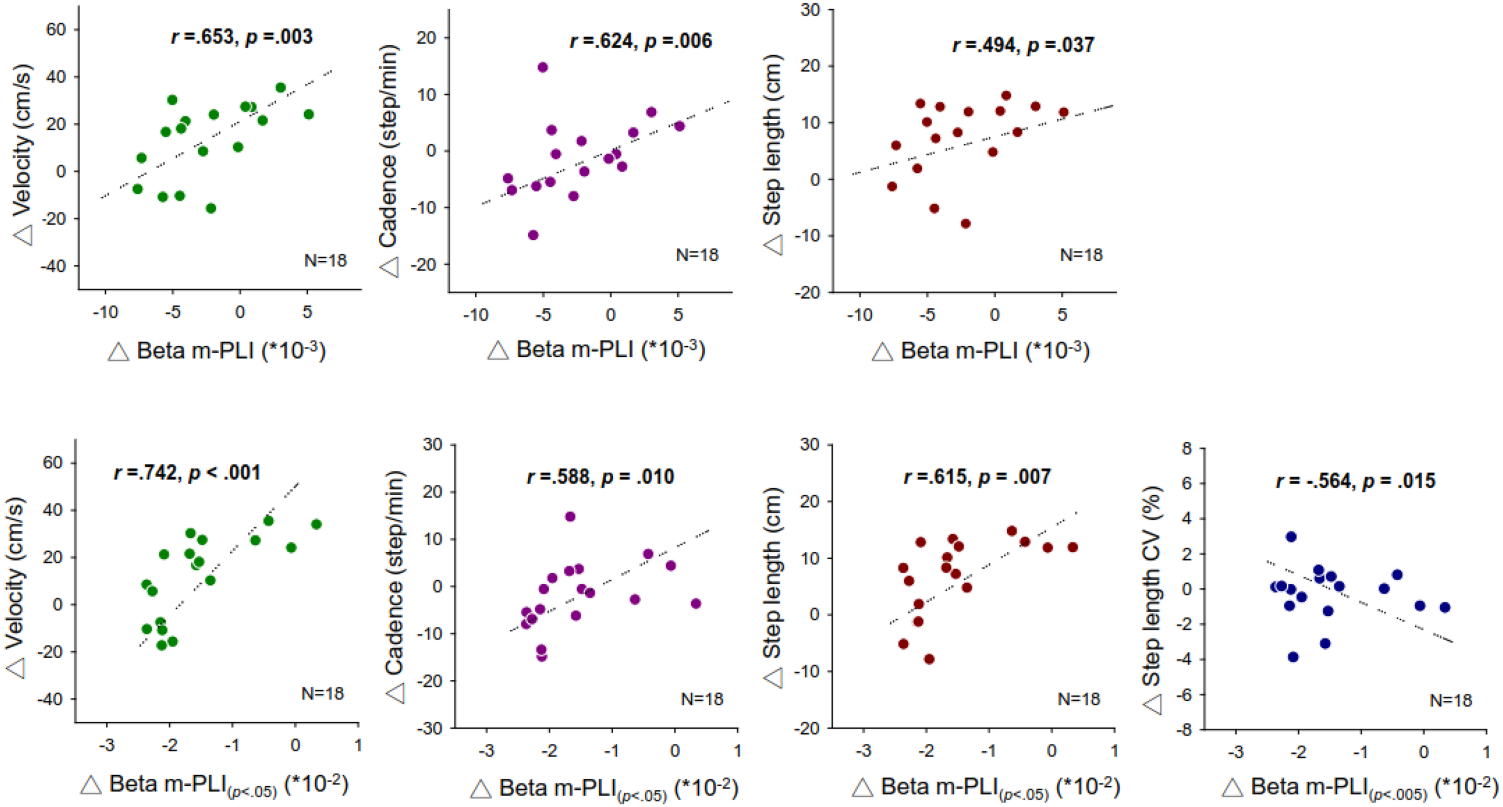
Plots of significant correlations in awareness-related change in the m-PLI of the beta band and gait parameters. m-PLI, mean phase-lag index.

For the correlation between walking performance and EEG connectivity, Figure 4 shows the scatter plots of awareness-related changes in the m-PLI of all electrode pairs in the beta band (△ Beta m-PLI) and gait parameters with significant correlations. The complete correlational results of △ Beta m-PLI and behavior parameters are documented in Supplementary Table 1. The △ Beta m-PLI positively correlated with awareness-related changes in gait velocity (△ velocity; *r* = 0.653, *p* = 0.003), cadence (△ cadence; *r* = 0.624, *p* = 0.006), and step length (△ step length; *r* = 0.494, *p* = 0.037). The awareness-related changes in the m-PLI of above-threshold connectivity from beta suppression pairs (△ Beta m-PLI_(*p* < 0.05)_) also positively correlated with awareness-related change in gait velocity (△ velocity; *r* = 0.742, *p* < 0.001), cadence (△ cadence; *r* = 0.588, *p* = 0.010), and step length (△ step length; *r* = 0.615, *p* = 0.007). Interestingly, the awareness-related change in the m-PLI of supra-threshold connectivity from beta suppression pairs (△ Beta m-PLI_(*p* < 0.005)_) a negatively correlated with awareness-related changes in step-length variability (△ step length CV; *r* = −0.564, *p* = 0.015). However, we found no significant correlation between awareness-related changes in the m-PLI of above-threshold connectivity from beta enhancement pairs (△ Beta m-PLI_(*p* < 0.05)_) and any awareness-related changes in behavior variables (*p* > 0.05).

## Discussion

Using the awareness strategy of taking big steps, individuals with PD and mild gait impairment were able to improve their gait performance for increased gait velocity and step length, concurrent with global suppression of beta connectivity at the cortical level. Changes in the m-PLI of the beta band between the AS and NAS conditions positively correlated with awareness-related changes in gait velocity, cadence, and step length, and negatively correlated with step-length variability. This study is the first to reveal an effect of stride awareness on suppressive modulation of beta inter-regional connectivity to improve gait performance in individuals with PD and mild gait impairment.

### Improved gait performance and functional connectivity with stride awareness

Our behavioral results are consistent with previous work that reported increased gait velocity and step length for individuals with PD using an awareness strategy (or attentional strategy) of taking big steps (Canning, 2005; Baker et al., 2007; Lowry et al., 2010; Lohnes and Earhart, 2011; Iansek et al., 2013). For single-task walking, the awareness strategy could improve step length in individuals with PD even in the off-medication state (Iansek et al., 2006). For dual-task walking, the awareness strategy could also lead to greater walking speed and step length for patients in the on-medication state (Canning, 2005; Baker et al., 2007; Lohnes and Earhart, 2011). The present study further indicates that the awareness strategy is effective for dual-task walking even in the off-medication state in PD with mild gait impairment. For the sake of convenience, awareness strategy-based dual-task walking is highly recommended to train individuals with PD in clinical practice and at home. The awareness strategy concept in this study reinforces the notion of LSVT-BIG therapy (Janssens et al., 2014; Peterka et al., 2020), which advocates repetitive practices with augmented movement amplitude, thereby reducing motor errors *via* better movement proprioception (Peterka et al., 2020). Without visual or verbal feedback, the attentional cuing of amplitude enlargement is hypothesized to add an integration of somatosensory inputs for neurological patients, despite the explanation being fairly speculative (Peterka et al., 2020).

The present study provides clear neural evidence of cortical reorganization when participants with PD focused on walking with big steps. The mean strength of beta functional connectivity was significantly reduced in the AS condition compared to the NAS condition (Figure 3; Table 3A). Beta oscillations from the basal ganglia serve as a communication channel for information transfer of cognitive and motor functions within the cortico-basal ganglia-thalamic circuits (Wagner et al., 2016; Singh, 2018; Schmidt et al., 2019). With reduced dopamine release in the striatum and subthalamic nucleus, the beta oscillations in PD are highly synchronized because the set-point of the beta oscillation networks is pathologically biased in these patients (Brittain and Brown, 2014). The degree of beta synchronization in PD is linked to poor movement quality and motor symptom severity, including akinesia and bradykinesia (Engel and Fries, 2010; Little and Brown, 2014). Levodopa intake or subthalamic-nucleus deep-brain stimulation can effectively alleviate exaggerated beta synchronization in return for superior control over the motor symptoms (Engel and Fries, 2010; Brittain and Brown, 2014; Prokic et al., 2019; Pauls et al., 2022). The reduction in the strength of inter-regional beta connectivity with the stride-awareness strategy seemed to restore walking capacity in PD, which is attributable to resetting the high set-point of beta oscillations to an appropriate lower level (Brittain and Brown, 2014; Little and Brown, 2014).

Using a needle-electrode EEG recording from the pedunculopontine nucleus, some previous studies argued that gait impairment could vary with modulations in a local region or synchronization network in the alpha band (Thevathasan et al., 2012; Fraix et al., 2013). However, in contrast to a reported decline in cortical alpha power with visual cue during walking (Stuart et al., 2021), we did not observe significant modulation of alpha connectivity during dual-task walking by participants with PD in the AS condition. The inconsistence speaks for distinct control processes of walking between internal and external cues in PD (Rochester et al., 2011). External cues appear to be more effective for gait dysfunction, especially gait velocity and variability (Rochester et al., 2011), and cortical alpha activity may be highly affected by visual information processes (Stuart et al., 2021).

### Additional insight into brain-gait correlation from awareness strategy

Notably, we found a positive correlation between awareness-related changes in the strength of the beta connectivity of suppressed connectivity pairs (△ m-PLI_(*p* < 0.05)_) and gait variables (△ velocity, △ cadence, and △ step length; Figure 4, Supplementary Table 1B). In addition, we found a negative correlation between awareness-related changes in the strength of the beta connectivity of suppressed connectivity pairs (△ m-PLI_(*p* < 0.005)_) and step-length variability (△ step length CV), whereas gait improvements did not depend on awareness-related changes in the strength of the beta connectivity of enhanced connectivity pairs (Supplementary Table 1C). Namely, stride awareness-related gait improvements can be predicted by variations in the mean strength of the inter-regional connectivity that is moderately or strongly suppressed with stride awareness in a statistical sense. Despite a decreasing trend in the beta connectivity strength with stride awareness, the patients who had a slight increase in the strength of the beta connectivity with stride awareness exhibited greater gait improvements than those who had decreased beta connectivity strength with stride awareness (Table 3).

**Figure 5:**
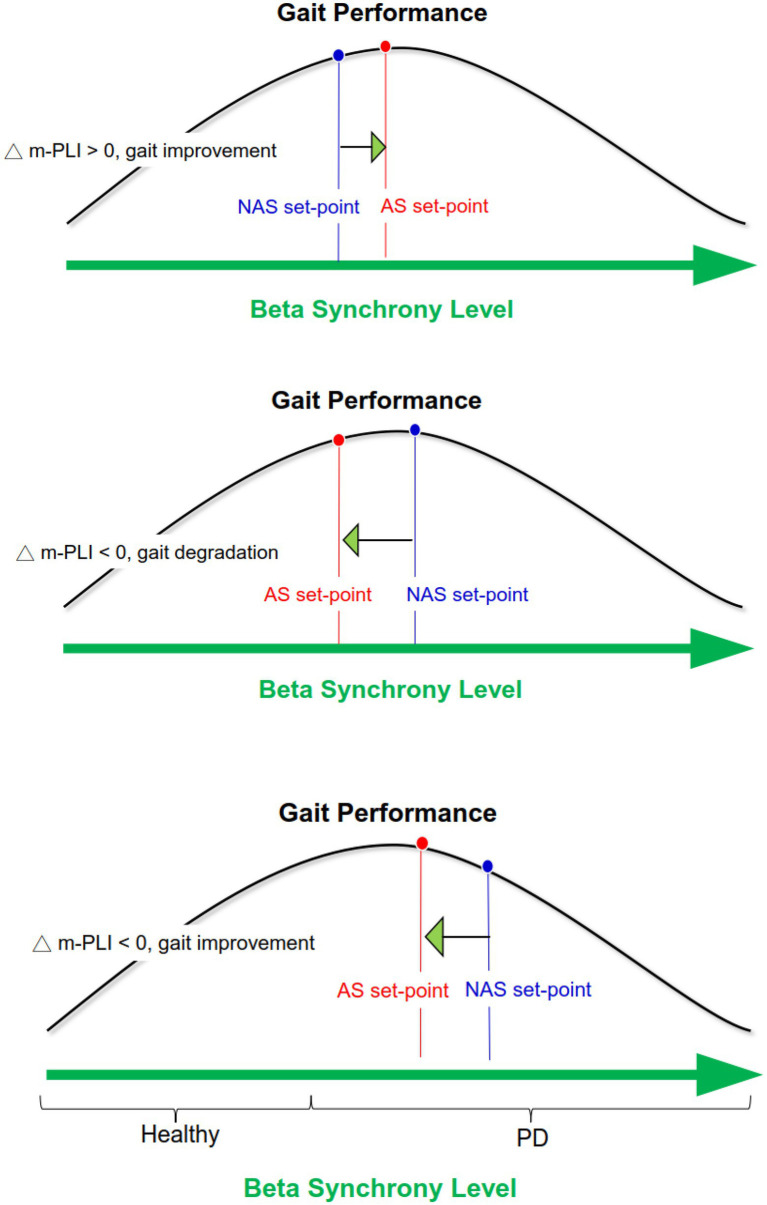
Schematic illustration of awareness-related changes in gait performance based on the U-shape relationship between system performance and the degree of beta synchrony.

Although the exact role of beta-band connectivity modulation on stride awareness-related gait improvement is not completely clear, the most likely explanation lies in the U-shape relationship between system performance and the degree of beta synchrony (Brittain and Brown, 2014; Little and Brown, 2014). According to the model, ensemble system performance is optimized under moderate beta synchrony, whereas ensemble system performance is worst under extremely low and high beta synchrony (Figure 5). In light of the global reduction in beta m-PLI (Table 3), it seems that beta synchrony suppressed to a lower set-point of the beta oscillation in the majority of our participants when they adopted a stride-awareness strategy during dual-task walking. Notably, some of the participants who had greater beta connectivity strength with the awareness strategy exhibited more remarkable improvement in their gait. For these participants, the set-point of beta synchrony in the NAS condition may be near the values of healthy individuals. Therefore, if stride awareness increases the set-point of beta synchrony, we can expect improvements in dual-task walking performance (Figure 5, upper plot). If the participants already have a moderately higher set-point of beta synchrony in the NAS condition, decreased beta connectivity strength in the AS condition would concur with degraded dual-task walking performance (Figure 5, middle plot). In contrast, if the participants have a relatively higher set-point of beta synchrony in the NAS condition, decreased beta connectivity strength in the AS condition would concur with improved dual-task walking performance, as in the exceptional cases in this study (Figure 5, lower plot).

Although several previous studies have shown that reducing excessive beta synchrony in PD is helpful in improving motor function (Prokic et al., 2019; Pauls et al., 2022), the phenomenon of greater beta connectivity strength with improved behavior while using an awareness strategy is somewhat parallel to the usage of an external cuing strategy in PD. With visuospatial cuing, individuals with PD demonstrate a faster reaction time for an action selection task, and this movement facilitation is associated with increased beta synchrony within the sensorimotor networks during preparation and execution of the externally cued action (Sparks et al., 2022). During a cued finger-tapping task, individuals with PD can better maintain a physiological rhythmic motor activity with increased beta functional connectivity between auditory and motor areas (Buard et al., 2019). Collectively, regardless of the form of the cuing strategy, motor facilitation is flexibly tuned to the network connectivity of beta dynamics (Leventhal et al., 2012; Mirzaei et al., 2017).

### Methodological concerns

To characterize the inter-regional connectivity, this study considered the PLI, which is comparatively immune to volume conduction artifacts, despite it may have a limitation in identifying the connectivity of non-stationaries (Cohen, 2015). Functional connectivity may be dynamically modulated in specific phases of the gait cycle (Gwin et al., 2011; Wang and Choi, 2020). Averaging the functional connectivity of the duration of whole gait cycles simplifies the time-dependent nature of functional connectivity during locomotion. Based on the time-dependent nature of functional connectivity, the roles of phase modulation in beta connectivity could be more comprehensive in adopting a stride awareness strategy for individuals with PD. However, the major technical barrier for dynamic EEG connectivity is the selection of sliding window length for a short period of gait cycles (Sahib et al., 2018). Next, relatively small sample size might lead to sampling bias of the entire contour, suggesting the need for a greater number of participants and different severity of gait impairment in individuals with PD for further investigation. In addition, with greater sample size, we could examine whether there are sex specific differences for awareness strategy or not. This study simply targeted PD with mild gait impairment because they may retain the ability to walk while performing a bimanual task. Therefore, whether the effect of a stride awareness strategy can be generalized to PD with relatively severe gait impairment requires further investigation. Despite the small sample size, our study is the first work to examine the effect of awareness strategy on dual-task walking in PD and to link the awareness-related change in walking performance to a specific form of brain functional connectivity. On the other hand, we did not compare stride-awareness effect between people with PD and healthy adults in the present study. Based on previous studies, shortened stride length is the most consistent finding of walking impairment in PD, particularly under dual-task walking conditions (Kelly et al., 2012), but shortened stride length is not necessary to be a specific gait symptom for healthy older adults (Fallahtafti et al., 2021; Hennah et al., 2021). For example, prominent dual-task interference on step length of healthy older adults may only be observed when walking along a narrow path (width = 40 cm), but not when walking along a wide path (width = 80 cm)(Hennah et al., 2021). Adopting an awareness strategy of focusing on “taking big steps” might not be an appropriate walking strategy for healthy older adults. Moreover, focusing on “taking big steps” is one of recommended walking strategies for individuals with PD (Nonnekes et al., 2019). Therefore, we only recruited individuals with PD for investigating the association between awareness strategy and related brain functional connectivity of dual-task walking control in the present study. Finally, for the definition of mild gait impairment, we only chose item 2.12 (walking and balance) and item 3.10 (gait) of MDS-UPDRS because these two items may reflect the situation of gait impairment under usual walking conditions for participants. We did not include item 3.11 (freezing of gait) and item 3.12 (postural instability) of MDS-UPDRS because the appearance of freezing of gait is often affected by time constraints, crowded places/narrow spaces, or anxiety state of people with PD (Nutt et al., 2011; Ehgoetz Martens et al., 2014), and item 3.12 (i.e pull test) may more likely to test the ability of standing balance for dealing with external perturbation rather than to test walking ability (Pérez-Sánchez and Grandas, 2019). In addition, the score of item 3.11 is 0 for all participants, and the mean score of item 3.12 for all participants is 1.06 ± 0.80 (range 0 to 2), indicating the participants did not has a symptom of freezing of gait and the ability of postural stability was between normal to mild impairment.

## Conclusion

For individuals with PD and mild gait impairment, an awareness strategy for taking big steps increased gait velocity and step length without undermining manual performance during dual-task walking. In clinical practice, individuals with PD and mild gait impairment are encouraged to focus on taking big steps during dual-task walking in order to enhance gait performance, even in the off-medication state. These performance benefits involve characteristics with modulation of the cortical beta networks. For individuals with PD and mild gait impairment, greater reduction in beta connectivity with the stride awareness strategy is associated with less gait improvement, and vice versa.

## Data availability statement

The raw data supporting the conclusions of this article will be made available by the authors, without undue reservation.

## Ethics statement

The studies involving human participants were reviewed and approved by National Taiwan University Hospital Research Ethics Committee. The patients/participants provided their written informed consent to participate in this study.

## Author contributions

Substantial contributions to the conception or design of the work; or the acquisition, analysis, or interpretation of data for the work. C-YH, Y-AC, R-MW, and I-SH: conception or design of the work. C-YH, Y-AC, and R-MW: acquisition. C-YH, Y-AC, and I-SH: analysis. C-YH and I-SH: interpretation of data. C-YH: funding acquisition. C-YH and I-SH: drafting the work or revising it critically for important intellectual content. C-YH, Y-AC, R-MW, and I-SH: final approval of the version to be published. All authors contributed to the article and approved the submitted version.

## Funding

This work was supported by a grant from the Ministry of Science and Technology, R.O.C. Taiwan (grant no. MOST 109-2314-B-002-115-MY3).

## Conflict of interest

The authors declare that the research was conducted in the absence of any commercial or financial relationships that could be construed as a potential conflict of interest.

## Publisher’s note

All claims expressed in this article are solely those of the authors and do not necessarily represent those of their affiliated organizations, or those of the publisher, the editors and the reviewers. Any product that may be evaluated in this article, or claim that may be made by its manufacturer, is not guaranteed or endorsed by the publisher.
