# Supplementary material for: Dual-task walking improvement with enhanced kinesthetic awareness in Parkinson’s disease with mild gait impairment: EEG connectivity and clinical implication
Source: Front Aging Neurosci. 2022 Nov 30;14:1041378. doi: 10.3389/fnagi.2022.1041378 (PMC9748616; doi:10.3389/fnagi.2022.1041378)
Supplement: Supplementary file 1 [file Table_1.DOCX]

**Supplementary Table 1.** **(A)** Spearman correlation of awareness-related changes in connectivity strength in beta band and dual-task performance. **(B)** Correlation of beta connectivity suppression (AS < NAS) with changes in dual-task performance. **(C)** Correlation of beta connectivity enhancement (AS > NAS) with changes in dual-task performance.

| **All connectivity pairs** | **△ Velocity**  (cm/s) | **△ Cadence**  (step/min) | **△ Step Length**  (cm) | **△ Step Length CV**  (%) | **△ Ring-touch Time** (%) |
| --- | --- | --- | --- | --- | --- |
| **△ m-PLI** | ***r* = .653, *p* = .003** | ***r* = .624, *p* = .006** | ***r* = .494, *p* = .037** | *r* = .187, *p* = .458 | *r* = −.209, *p* = .404 |

**(A)**

| **Suppression pairs** | **△ Velocity**  (cm/s) | **△ Cadence**  (step/min) | **△ Step Length**  (cm) | **△ Step Length CV**  (%) | **△ Ring-touch Time** (%) |
| --- | --- | --- | --- | --- | --- |
| **△ Beta m-PLI _(_*_p_* _< .05)_** | ***r* = .742, *p* < .001** | ***r* = .588, *p* = .010** | ***r* = .615, *p* = .007** | *r* = −.098, *p* = .699 | *r* = −.387, *p* = .113 |
| **△ Beta m-PLI _(_*_p_* _< .005)_** | *r* = −.137, *p* = .587 | *r* = −.096, *p* = .705 | *r* = −.020, *p* = .938 | ***r* =** −**.564, *p* = .015** | *r* = −.102, *p* = .687 |

**(B)**

| **Enhancement pairs** | **△ Velocity**  (cm/s) | **△ Cadence**  (step/min) | **△ Step Length**  (cm) | **△ Step Length CV**  (%) | **△ Ring-touch Time** (%) |
| --- | --- | --- | --- | --- | --- |
| **△ Beta m-PLI _(_*_p_* _< .05)_** | *r* = −.366, *p* = .135 | *r* = −.225, *p* = .369 | *r* = −.273, *p* = .272 | *r* = −.416, *p* = .086 | *r* = .018, *p* = .945 |

**(C)**

*Notes*: AS = awareness strategy; NAS = non-awareness strategy; m-PLI = mean phase-lag index; CV = coefficient of variation;

m-PLI _(_*_p_* _< .05)_ = mean PLI from the electrode pairs above-threshold (*p* < .05); m-PLI _(_*_p_* _< .005)_ = mean PLI from the electrode pairs supra-threshold (*p* < .005)
